# Stem/progenitor cells in fetuses and newborns: overview of immunohistochemical markers

**DOI:** 10.1186/s13619-021-00084-6

**Published:** 2021-07-05

**Authors:** D. Fanni, C. Gerosa, C. Loddo, M. Castagnola, V. Fanos, M. Zaffanello, G. Faa

**Affiliations:** 1 Division of Pathology, University Hospital San Giovanni Di Dio, via Ospedale, 54, Cagliari, Italy; 2 Department of Biology, College of Science and Technology, Temple University, Phidelphia, USA; 3 Neonatal Intensive Care Unit, Department of Surgical Sciences, University of Cagliari, Cagliari, Italy; 4 Laboratory of Biochemistry and Metabolomics, IRCCS Fondazione Santa Lucia, Rome, Italy; 5 Department of Surgical Sciences, Dentistry, Gynecology and Pediatrics, University of Verona, Piazzale Stefani, 1, I-37126 Verona, Italy

**Keywords:** Fetus, Immunohistochemical analysis, Newborn, Progenitor cells, Stem cells

## Abstract

Microanatomy of the vast majority of human organs at birth is characterized by marked differences as compared to adult organs, regarding their architecture and the cell types detectable at histology. In preterm neonates, these differences are even more evident, due to the lower level of organ maturation and to ongoing cell differentiation. One of the most remarkable finding in preterm tissues is the presence of huge amounts of stem/progenitor cells in multiple organs, including kidney, brain, heart, adrenals, and lungs. In other organs, such as liver, the completely different burden of cell types in preterm infants is mainly related to the different function of the liver during gestation, mainly focused on hematopoiesis, a function that is taken by bone marrow after birth. Our preliminary studies showed that the antigens expressed by stem/progenitors differ significantly from one organ to the next. Moreover, within each developing human tissue, reactivity for different stem cell markers also changes during gestation, according with the multiple differentiation steps encountered by each progenitor during development. A better knowledge of stem/progenitor cells of preterms will allow neonatologists to boost preterm organ maturation, favoring the differentiation of the multiple cells types that characterize each organ in at term neonates.

## Background

In recent years, many different types of stem/progenitor cells have been discovered in the human body, all characterized by their ability to self-renew and differentiate towards the different cell types that put the spotlight on the complex architecture of each human organ. (Schmelzer et al., 2019) At histology, under the microscope, stem/progenitor cells do not show any peculiar finding and their identification, when exclusively based on morphology, is very difficult or impossible.

In order to identify the multiple stem/progenitor cells detectable in human tissues, many immunohistochemical markers have been proposed during the years. Their use allowed the characterization of undifferentiated self-renewing stem cells, their initial differentiation into precursors, and the different phases of their progressive differentiation from progenitors towards the typical cell types that characterize the mature human tissues. (Zakrzewski et al., 2019) The strategy for the identification of stem/progenitors in human tissues has been based on the use of multiple antibodies. The expression of markers such as OCT4, Sox2, NANOG, SSEA4, TRA 1–60, and TRA 1–81 should be utilized as pluripotency markers of embryonal stem cells (ESC). The differentiation potential of pluripotent/stem cells toward the three-germ layer should be analyzed by RT-PCR or by the expression of key markers of lineage commitment such as alpha-feto-protein (AFP) (endoderm), desmin (mesoderm), and CRABP2 (ectoderm). The differentiation potential of stem cells should be also based on the use of antibodies against cytokeratin 17 (endoderm), desmin (mesoderm), and vimentin (ectoderm). (Kim et al., 2014)

Some transcription factors, including Oct4 and Nanog, are typically expressed by embryonic stem cells, being associated with maintenance of embryonic stem cells in an undifferentiated status. In recent years, very small embryonic-like pluripotent stem cells have been reported in adult mouse tissues, all characterized by the small size (3–6 μm) and, as a consequence, they are rarely identifiable at histology. (Ratajczak et al., 2012) In immunohistochemistry, these stem/pluripotent cells were characterized by the expression of Oct-4, SSEA-1 and Sca-1. (Kim et al., 2014)

## Main Text

## Aims

To our knowledge, there is a lack of practical guidance for the identification of stem / progenitor cells in human fetuses and newborns.

With this review we report the most recent data regarding the immunohistochemical markers suitable for their identification and in situ detection of stem progenitor cells in the vital human organs during gestation and at birth, in order to allow perinatal pathologists to identify pluripotent cells in embryo, fetal and newborn tissues.

## Methods

We searched the relevant articles in the literature using online databases (PubMed and Scopus) that regard steam cells in fetus (amniotic, umbilical, breast, breastmilk, thyroid, cardiac, pulmonary, cutaneous, pancreatic, adrenal, intestinal, liver, brain, and endometrial stem cells in fetus). The most important findings of all these putative stem cells niches will be summarized. Heart, lungs, kidneys, liver, gut, adrenal glands were included in this study because they are vital organs; amniotic membranes, umbilical cord breast and breast milk are important for embryo, fetus and newborn development and nutrition. Thyroid, skin, pancreas and endometrium were added.

For each section, we report related figures. For each tissue, section of the thickness of 3–4 μm were obtained. The morphological study of samples was mainly based on the histological study of hematoxylin and eosin-stained sections. Tissue sections were accurately scanned at high power (400–630 X) by two pathologists (FD and GC), in order to detect the presence of possible stem cell niches in the developing tissue. In each sample, the immuno-histochemical markers were based on data derived from the literature (Fanni et al., 2015a).

## Results

### Amniotic stem cells

The amnion membrane is developed from embryo-derived cells, and amniotic cells exhibit multi-differentiation potentials. Amniotic membranes contain pluripotent, multipotent, precursor, and differentiated cells (Miki et al., 2007). Amniotic membranes contain epithelial stem/progenitor cells with unique CD phenotype expressing a number of intracellular embryonic stem cells makers and mesenchymal stem cells with unusual CD phenotype expressing a different set of intracellular embryonic stem cells markers (Farhadihosseinabadi et al., 2018). The biological properties of these cell populations have been only recently characterized, due to some studies focused on the identification of amniotic stem cell subpopulations and their differentiation potential.

Human fetal mesenchymal stem cells can be isolated from the amniotic membrane (AM-hMSCs) by enzymatic digestion. Two main subpopulations were isolated: CD44+ / CD73+ / CD105+ and CD44+ / CD73+ / CD105-. The analysis of the expression of pluripotency-associated markers showed positive expression of SOX2, SOX3, PAX6, OCT3/4, and NANOG in the CD105+ and CD105- cell subpopulations (Leyva-Leyva et al., 2013). Another study carried out on amnion-derived cells by flow cytometry revealed the expression in amnion epithelial cells of CD133, CD 271, and TRA-1-60, whereas mesenchymal cells expressed CD44, CD73, CD90, and CD105. In the same study, immunohistochemistry showed that both cell types express Oct3/4, Sox2, Klf4, and SSEA4 (Leyva-Leyva et al., 2013).

AM-hMSCs, extracted from the amniotic membrane show the following phenotype: CD105+, CD73+, CD29+, CD44+, CD166+. This phenotype is consistent with the phenotype reported in bone marrow derived from mesenchymal cells (Alviano et al., 2007). In the same study, AM-hMSCs, were shown to undergo in vitro chondrogenic, myogenic, adipogenic and osteogenic differentiation.

Recent studies lay stress on the ability of human amniotic stem/progenitors to differentiate towards multiple cell types, representing a useful tool in many fields of the regenerative medicine. In particular, amniotic mesenchymal stem cells may differentiate into fibroblasts that might be utilized for the regeneration of the anterior cruciate ligament of the human knee. (Differentiation of Human Amniotic Mesenchymal Stem Cells Into Human Anterior Cruciate Ligament Fibroblast Cells by In Vitro Coculture - PubMed, 2020) Moreover, amnion-derived mesenchymal stem cells have been shown to promote osteogenic and angiogenic differentiation of human adipose-derived stem cells. (Zhang et al., 2017)

Given the high ability of AM-hMSCs to differentiate towards multiple cells type, isolation of pluripotent stem cells has been proposed, in recent years, as an alternative source for tissue regeneration, able to give rise to a new area in regenerative medicine and for either autologous or allogenic transplantation (Srivastava et al., 2018).

Stem cells extracted from the amniotic fluid, during years, have been utilized in studies aimed to improve tissue regeneration in multiple setting, including kidney regeneration (Perin et al., 2007), myocardial infarction (Bollini et al., 2011), necrotizing enterocolitis (Zani et al., 2014), lung regeneration (Carraro et al., 2008) and neoangiogenesis (Lloyd-Griffith et al., 2015). Recently, extracellular vesicles derived from amniotic fluid stem cells, have been shown to mediate pro-angiogenic and immune-modulatory effects in the treatment of Alport syndrome (Antounians et al., 2019).

Stem cells from the amnion have been classified in different progenitor cells populations by an international workshop (Parolini et al., 2008). These amniotic stem cell populations include human amniotic epithelial cells (h-AECs), human amniotic mesenchymal stromal cells (h-AMSCs), human chorionic mesenchymal stromal cells (h-CMSCs) and human chorionic trophoblastic cells (h-CTCs) (Miki et al., 2005) (Alviano et al., 2007) (Soncini et al., 2007).

According with their different origin, amnion stem cells express embryonic specific markers (SSEA3, SSEA5, TRA-1-60, TRA-1-81) and mesenchymal markers (CD105, CD90, CD73, CD44, CD29, CD13, CD10, CD166, CD117) (De Coppi & Atala, 2019).

Moreover, two populations of stem/progenitor cells have been identified in the amniotic fluid: the amniotic fluid: the amniotic fluid mesenchymal stem cells (AFMSCs) and the amniotic fluid stem (AFS) cells. All these stem cell populations have specific advantages and disadvantages for therapeutic purpose and represent an attractive resource for the treatment of the multiple congenital and acquired diseases (De Coppi & Atala, 2019) (Table 1).

**Table 1:** most useful markers for the immunohistochemical identification of organ progenitors

Subtypes of stem cells	Tissue	Stem cell markers
Amniotic stem cells markers	Mesenchymal	CD105+; CD44+; CD73+; CD90+; CD29+; CD166+; CD13+; CD10+ (22); CD117+ (22)
	Epithelial	CD105-; NANOG+; CD133+; CD 271+; TRA-1-60+
	Mesenchymal and epihelial	CD44+; CD73+; SOX2+; SOX3+; PAX6+; OCT3/4+; KLF4+; SSEA4+
	Embryonal	SSEA3+ (22); SSEA5+ (22); TRA-1-60+ (8) (22); TRA-1-81+ (22)
Umbilical cord stem cells markers	Mesenchimal	α-SMA+ (27); VCAM+ (27); CD44+ (29); CD73+ (29); CD105+ (29); CD90 (29); CD45- (29); CD34- (29); CD19- (29); HLA-DR- (29); CD11b - (29)
	Endothelial	CD34+ (31); CD133+ (31); VEGFR+ (31)
Breast stem cells markers and breast milk stem cells markers	Breast stem cells	CD44+ (33); CD24- (33); ALDH1- (33)
	Breast milk stem cells	Oct4+ (38); NANOG+ (38); CD49f+ (38); nestin+ (39) (41); ESRRB+ (40); CK5+ (40); CK14+ (40); α-lactalbumin+ (40); CD44+ (41) (42); CD29+ (41); Sca-1+ (41); vimentin+ (41); smooth muscle actin+ (41); Ki67+ (42)
Thyroid stem cells markers	Human	CD44+; POU5F1+; nestin+; Oct- 4 +; GATA-4+; HNF4α+
	Mouse and rat	VEGF-A+ (mouse); NANOG+ (rat); ABCG2+ (rat); GATA4+ (rat)
Renal stem cells markers		CD133+; CD24; CD44+; thymosin beta-4+; MUC-1+; CD10+; WT1+
Cardiac stem cells markers		Sca-1+; CD31+; CD38+; GATA-4+; MEF 2C+; TEF-1+; WT1+; Wnt1+; CD44+; ISL1+ (70); c-kit- (64); CD34- (64); CD45- (64)
Pulmonary stem cells markers		TTF1+ (73)
	Epithelial precursors	SOX2+
	Mesenchyme	WT1+
Cutaneous stem cells markers	Melanocytes	CD20+; CD133+
	Hair follicles	CK15+; nestin+
Pancreatic stem cells markers		PDX1+; Ptf1-alpha+; SOX9 +
Adrenal stem cells markers		NCAM+; CD117+; NSE+; PDGFr-alpha+; Synaptophysin+; Hepatocyte growth factor+; alpha-feto-protein+; Pbx1+; SF1+
Intestinal stem cells markers		Lgr5+; Wnt+; BMP+; Notch+; EGF+; p-TEN+; p-AKT+; Fgfr3+; CD44 +
	Paneth cells	CD24+
Liver stem cells markers	Biliary progenitor	CK19+; CK7+
	Multipotent undifferentiated hepatic stem/progenitors	NCAm+; CD133+; SOX9+; SOX17+; FOXA2+; Ck 8/18+
	Hepatoblasts	albumin+; CYPA4+; CYPA7+
	Committed hepatic progenitors	albumin+; glucose-6-phosphate+; CK19+; α-fetoprotein+
Brain stem cells markers	Primary neuroepithelium	Nestin+
	Radial glia	Nestin+; vimentin+; WT1+
	Brain stem cells	SOX2 (103)
Endometrial stem cells		CD44+; CD90+; CD105+; OCT4+; c-KIT (CD117)+; CD34+; bcl2+; CD146+; PDGF-Rbeta+; ISL1

### Umbilical cord stem cells

The human umbilical cord tissue surrounding the umbilical vein and the two umbilical arteries, generally known as the Warthon jelly, is a rich source of two main subtypes of stem cells: i) the cord blood stem cells (Çil et al., 2017) and ii) the cord tissue stem cells. (Harris, 2013) Both types of stem cells are characterized by their easy accessibility, also by a special flexibility, that enables them to easily adapt to a patient’s body during transplant. (Kiernan et al., 2017) Thanks to these features, the therapeutic potential of cord stem cells is generally considered to be particularly vast. (Sun et al., 2017) The subendothelial layers of the umbilical vein have isolated mesenchymal stem cells. Their phenotype is characterized by immunoreactivity for alpha- smooth muscle actin (alpha-SMA), vascular cell adhesion molecule (VCAM) and by the deposition of fibronectin and collagen (Romanov et al., 2003). The expression of alpha-SMA and cell adhesion molecules including VCAM, suggests that these umbilical cord-derived stem cells should be considered mesenchymal progenitors with multilineage differentiation potential (Jaiswal et al., 2000). A study based on flow cytometric analyses has better defined the phenotype of human umbilical mesenchymal stem cells, that are characterized by reactivity for CD44, CD73, CD105 and CD90, whereas CD45, CD34, CD19, HLA-DR, and CD11b are not expressed (Wu et al., 2017).

A recent review on umbilical cord stem cells clearly identified the human umbilical cord as an attractive source for autologous and allogenic stem cells, which are ethically noncontroversial and readily aviable (Alatyyat et al., 2020). In the same review, multiple population of stem cells are identified in the umbilical cord, including endothelial progenitor cells (CD34+, CD133+, VEGFR+) (Lee et al., 2014), hematopoietic stem cells, epithelial stem cells, mesenchymal stem cells and induced pluripotent stem cells (Alatyyat et al., 2020) (Table 1).

#### Breast stem cells

The breast is a peculiar organ in that it fully matures during pregnancy and lactation, when the mammary gland undergoes complete remodeling of its epithelial and stromal components, finalized to support the secretion of milk and its delivery to the breastfeeding infant. (Twigger et al., 2013a) Mammary stem cells are responsible for the changes that occur in the breast during this period, which can be repeated several times during the life of a woman. The normal epithelium of the breast is organized in a cellular hierarchy, characterized by an estrogen receptor negative (ER-) stem cell that originates both ER+ and ER- progenitors, which differentiate into the luminal and the myoepithelial/basal epithelium. The immunophenotype of mammary stem cells is given by the expression of CD44, in the absence of any reactivity for CD24 (Cabuk et al., 2016) and of aldehyde dehydrogenase I (ALDH1), a detoxifying enzyme that promotes the oxidation of intracellular aldehydes. Recently, the mammary gland has been shown to contain different types of stem/progenitor cells. In the postnatal unperturbed mammary glands, both luminal and myoepithelial lineages contain long-lived unipotent stem cells that display renewing capacities, being able to clonally expand during morphogenesis, eventually undergoing massive expansion during pregnancy. (Van Keymeulen et al., 2011) A recent study carried out in mice, through clonal cell-fate mapping studies, evidenced the existence in the adult mammary gland of bi-potent mammary stem cells, able to differentiate along the two primary lineages of the mammary gland epithelium, the inner luminal and the outer myoepithelial cell layer. These data support a model in which both stem and progenitor cells drive morphogenesis during puberty, whereas bi-potent mammary stem cells coordinate ductal homeostasis and remodeling of the mouse mammary gland. (Rios et al., 2014) The existence of multipotent mammary stem cells and at least of two distinct luminal progenitor types has been recently confirmed in the human breast. The existence of a complex stem cell compartment within the mammary gland, defining a functional stem cell hierarchy in human breast, has been hypothesized in recent years. (Fu et al., 2014) There is accumulating evidence for the existence of a heterogeneous mammary stem cell compartment, comprising fetal stem cells, slowly cycling cells, long-term and short-term repopulating cells. Moreover, diverse luminal progenitor subtypes have been identified in the human mammary gland (Visvader & Stingl, 2014).

#### Breast milk stem cells

Human milk contains numerous maternal breast-derived stem cells that can differentiate towards cells of all the three germ layers. The fate of human breast milk stem cells is unknown, but recent data in experimental animals suggest that a part of them is not lost in the breastfeeding child. Maternal stem cells ingested by newborn mice have been shown to adhere to the gastric wall and penetrate into the stomach wall, eventually reaching the liver and other organs of the lactating newborn. (Twigger et al., 2013b) Mouse breast milk stem cells where characterized, at immunohistochemistry, by reactivity for the stem cell markers Oct4, NANOG and CD49f. In another study on stem cells in the human milk, nestin was identified as the best marker for their identification, suggesting that human breastmilk should be considered as a readily available and non-invasive source of stem/progenitor cells. (Cregan et al., 2007) These experimental data suggest that breast milk stem cells may integrate into multiple organs of the newborn and differentiate into multiple functional cell types all around the neonatal body, actively participating to the postnatal development and persisting throughout life. Stem cells in human breast milk have been recently shown to have pluripotent features, being able to differentiate into many cell types, including neural cells. (Twigger et al., 2013a) A recent study on the gene expression profile of breast milk stem cells showed that, in these cells, genes involved in stem cell regulation and milk production were closely associated, being responsible for the expression of stem cell markers such as ESRRB and CK5, myoepithelial markers such as CK14, and lactocyte markers, including α-lactalbumin. (Twigger et al., 2015) Mesenchymal stem cells isolated from the human milk express of CD44, CD29, Sca-1, nestin, vimentin and smooth muscle actin, while do not express CD33, CD34, CD45 and CD73. (Eirew et al., 2008) Recent data from our Lab, showed the expression of CD44 and Ki67 in stem cells of the human breast milk (Pichiri et al., 2016) (Table 1).

#### Thyroid stem cells

Three types of fetal thyroid cells exist in fetuses or young children: thyroid stem cells (TSCs) and two progenitor cells including thyreoblasts and prothyrocytes. (Takano, 2014) In thyroid tumors, including papillary thyroid carcinoma, CD44 resulted the best marker for the identification of thyroid stem cells, that also expressed POU5F1. (Ahn et al., 2014) In another immunohistochemical study, carried out in anaplastic thyroid carcinoma, nestin appeared the most specific marker for stemness. (Liu & Brown, 2010) A major role in thyroid development has been recently assigned to endothelial precursors. In developing mouse thyroid, epithelial production of VEGF-A has been shown to be necessary for endothelial cell precursors’ recruitment and expansion. In the adult human thyroid gland, the stem cell marker Oct- 4 and the early endodermal markers GATA-4 and HNF4alpha have been shown to represent the typical markers of thyroid stem cells. (Thomas et al., 2006) In a rat thyroid cell line, 3 stemness genes, NANOG, ABCG2 and GATA4, were expressed (Shimasue et al., 2015) (Table 1).

#### Renal stem cells

**Fig. 1:**
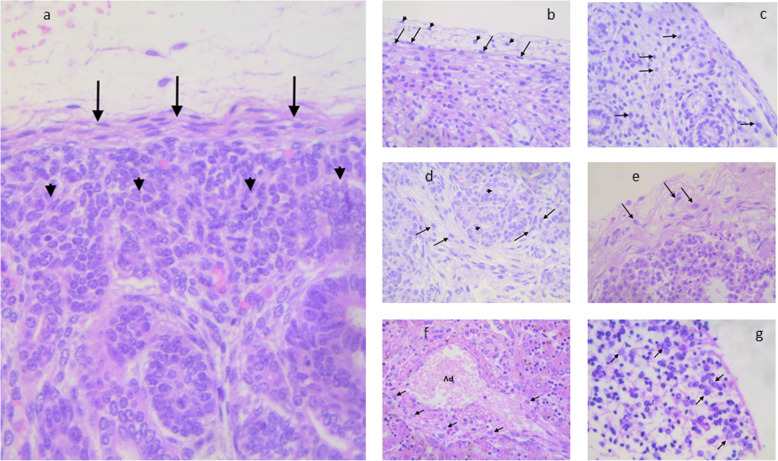
**a** Intracapsular (arrows) and subcapsular (arrowheads) renal mesenchymal stem cells. Fig. 1**b** - Cardiac progenitors in the subepicardial (arrows) and intraepicardial (arrowheads) zones. Fig. 1**c** - Undifferentiated mesenchymal pulmonary precursors (arrows) embedded in a loose myxoid stroma. Fig. 1**d** – Mesenchymal precursors (arrows) in a myxoid stroma surronding developing esocrine ducts (arrowheads). Fig. 1**e** – Mesenchymal stem cells (arrows) embedded in the adrenal capsule. Fig. 1**f** – Mesenchymal precursors (arrows) scattered at the periphery of immature portal tracts. PV= portal vein branch. Fig. 1**g** – Neuronal precursors (arrows) in the immature brain cortex.

The existence of renal stem/progenitor cells, marked by regenerative potential toward glomerular and tubular lineages has been reported in recent years by numerous authors in the mature adult kidney. (Angelotti et al., 2012) These adult stem/progenitors have been defined at immunohistochemical level by the co-expression of CD133 and CD24. The preterm kidney is characterized by a huge amount of metanephric mesenchymal stem/progenitors cells, which are aggregated in the subcapsular strip that, in H&E-stained sections, appears as a blue zone, due to the high nuclear/cytoplasmic ratio of renal stem cells (Fig. 1a). (Faa et al., 2013) The stem cell niches in the fetal and in the preterm kidney were formed by multiple cells types, including the ureteric bud tip cells and the surrounding induced cap mesenchymal cells. (Gerosa et al., 2016) Further studies identified multiple stem cell niches in the preterm kidney localized in different renal compartments including the capsule, the hilum, the sub-capsular nephrogenic zone, Bowman’s capsule, the cortical and medullary interstitium. (Fanni et al., 2015a) At immunohistochemistry, renal stem cells were reactive for CD44, (Fanni et al., 2013) thymosin beta-4, (Nemolato et al., 2014) MUC-1, (Fanni et al., 2011a) (Fanni et al., 2012) CD10 (Faa et al., 2012a) and WT1. (Ambu et al., 2015) (Fanni et al., 2011b) (Faa et al., 2012b) (Sanna et al., 2015) The preterm kidney appears to be a source of stem cells, that might be utilized in the next future. Physiological regenerative renal medicine is focused on prolonging nephrogenesis in all preterm neonates in order to escape oligonephronia and reduce the susceptibility to develop kidney disease later in life (Faa et al., 2015) (Fanos et al., 2015) (Table 1).

#### Cardiac stem cells

The ability to identify and focus on stem/progenitors in the human heart has been introduced in recent years by many researchers, with the potential to promote endogenous myocardial regeneration and prevent congestive heart disease (Hughes, 2002). In the human newborn, cardiac stem/progenitors may be easily identified in the sub-pericardial zone (Fig. 1b). Stem cell antigen-1 (Sca-1) has been first identified as a marker useful for identifying cardiac stem/progenitor cells in the adult human heart, Sca-1+ cells being able to replenish the cardiomyocyte population and generate coronary vessels. (Matsuura et al., 2004) Sca-1+ cells were identified as small interstitial cells and the phenotype of cardiac precursors has been better clarified. Cardiac Sca-1+ cells typically co-express platelet-endothelial adhesion molecules, including CD31 and its receptor CD38 and may express cardiogenetic transcription factors including GATA-4, MEF 2C, TEF-1. Cardiac progenitors do not express typical hematopoietic stem cell markers, including CD45, CD34, C-kit/CD117, GATA-2, Lmo2, and TAL1/Slc. Moreover, cardiac Sca-1+ cells do not express Nkx2.5, nor cardiac genes or endothelial progenitor cell markers such as Flk-1 and Flt-1. (Oh et al., 2003) The typical phenotype of cardiac progenitors (Sca-1+, CD31+, CD38+, CD34-, CD45-) allows their differentiation from multipotent muscle cells, that are characterized by coexpression of Sca-1 and CD34, a sialylated transmembrane glycoprotein. (Torrente et al., 2001) A novel cardiogenic precursor marked by expression of the transcription factor Wilms tumor-1 (Wt1) and located within the epicardium has been recently identified. *Wt1*^*+*^ pro-epicardial cells arise from progenitors that express Nkx2–5 and Isl1, suggesting that they share a developmental origin with multipotent *Nkx2–5*^+^ and *Isl1*^+^ stem/progenitor cells. These data identify *Wt1*^*+*^ epicardial cells as previously unrecognized cardiomyocyte progenitors, and suggest the use of this marker for their identification in the mature heart. (Zhou et al., 2008) A population of cardiac precursor cells has been identified from postnatal mouse hearts using isl-1 transcription factor as a cell marker. (Laugwitz et al., 2005) These cells are c-kit– and SCA-1–negative but are capable of differentiation into cardiomyocytes with electrical and contractile properties. Finally, another population of cardiac precursor cell has been studied in the developing and adult heart, able of proliferating and differentiating into cardiac and hematopoietic lineages in vitro. These cells were identified on the basis of expressing Abcg2, an ATP-binding cassette transporter, rather than by detection of surface markers. (Martin et al., 2004) On the basis of these data, the hypothesis is emerging on the existence of multiple pools of stem/progenitor cells in the adult heart, that might dramatically change the traditional view of the adult heart as a post-mitotic organ without any regenerative capacity. (Carvalho, 2010) The fetal and neonatal human heart is a precious model for the study of cardiac stem cells, due to the high number of progenitors detectable in any neonatal heart and to the presence of stem cell niches in the sub-pericardial zone. In a recent study from our group, carried out in neonatal hearts, CD44, ISL1, WT1 and Wnt1 were indicated as the most useful markers for the immunohistochemical identification of cardiac progenitors (Faa et al., 2016) (Table 1).

#### Pulmonary stem cells

The human lung is classified, from the regenerative point of view, as a conditionally renewing tissue, due to its very low turnover in basal conditions contrasting with its high regenerative abilities in case of lung injury. (Bertoncello & McQualter, 2013) The developing human lung represents a fascinating model for the study of lung progenitor cells, due to the high number of stem cells identifiable even in routine H&E-stained histological specimens (Fig. 1c). In a recent study from our group, stem/progenitors were identified in the neonatal lung in two main locations: in the interstitial septa and in close proximity of the pleura, where they were aggregated in stem cell niches. (Fanni et al., 2016a) In the same study, at immunohistochemistry TTF1 was reported as the highest nuclear marker of tip cells of the branching tubules, and of the sub-pleural stem cell niches, whereas SOX2 reactivity was restricted to the epithelial precursors of the proximal tubular structures. The developing pulmonary mesenchyme surrounding the branching immature bronchioles, was marked by WT1. These data taken together suggest the presence of multiple stem/progenitors in the newborn lung, that appears as a source of pulmonary stem cells that might be utilized for the regenerative therapy of multiple diseases, including bronchopulmonary dysplasia (Monz et al., 2016) (Table 1).

#### Cutaneous stem cells

Cells with stem-cell markers and features have recently been identified in melanoma tissues and cell lines. Melanoma stem-like cells possess self-renewal capacity, high tumorigenicity, and ability to differentiate into various cell lineages, including melanocytes. Two main subpopulations of melanoma-initiating cells have been distinguished by immunohistochemistry: CD20(+) and CD133(+). Whether these are distinct or overlapping populations is currently under investigation. Ongoing studies are dissecting and characterizing the hierarchy of these subpopulations within a malignant lesion. Understanding these and the dynamics of clonal dominance will aid in the development of novel therapeutic strategies. (Zabierowski & Herlyn, 2008) In the newborn skin, the developing hear follicles have been proposed as the putative location of cutaneous stem/progenitor cell niches. Recently, skin-derived stem cells have been better defined as a heterogeneous population of stem cells that show multipotency and can differentiate into neurons, glia, fibroblasts, muscle cells, adipocytes, osteoblats, chondroblasts, and pancreatic endocrine cells. Moreover, multipotent stem cells located in the hair follicles have been shown to derive from embryonic migratory neural crest or mesoderm cells. (Ge et al., 2016) Both cytokeratin (CK) 15 and nestin have been reported as follicular stem cell markers (Mahalingam et al., 2010) (Table 1).

#### Pancreatic stem cells

Pancreas originates from two buds emerging from the primitive foregut around the 3-4th week of gestation in humans. Around the 7th week, fusion of the two buds occurs, followed by the insertion of endocrine progenitors in the ductal epithelium context. This complex evolutionary frame well justifies the complexity of the pancreas structure during development, characterized by the contemporary presence of multiple stem/precursor cells in the fetal and the neonatal human pancreas (Fig. 1d). (Zhou et al., 2007) The pancreatic and duodenal homeobox 1 (PDX1) is expressed by pancreatic stem/precursors, being essential for the early specification of embryonic pancreas. (Bernardo et al., 2009) The expression of PDX1 is paralleled by the expression of the pancreas-specific transcription factor 1-alpha (Ptf1-alpha), that is considered a key factor in the signaling network that characterizes the early development of the human pancreas. (Wandzioch & Zaret, 2009) The pancreatic specification of human pluripotent stem cells is regulated by WNT and TGF-beta family members. (Nostro et al., 2011) SOX9 is expressed by pancreatic progenitors, being required for maintenance of the pancreatic progenitor cell pool. (Seymour et al., 2007) In a recent study from our group, the stem cell niche of the fetal and neonatal human pancreas was found at the periphery of the developing pancreas, in the subcapsular zone. Pancreatic stem/progenitors of the fetal and newborn pancreas showed a large oval nucleus and were enveloped by a loose myxoid stroma, in close proximity to the pancreatic capsule (Locci et al., 2016) (Table 1).

#### Adrenal stem cells

In recent years, the necessity to obtain adrenal stem cells for the therapy of adrenal insufficiency has rejuvenated the research on adrenal stem cells. (Ruiz-Babot et al., 2015) A recent article focused on adrenal stem cell niches in the human adrenal glands identified the stem cell niches in the adrenal cortex. In the newborn, the stem cell niches appear as nests of undifferentiated small cells, occasionally centered by a thick capillary (Fig. 1e). At immunohistochemistry, adrenal stem/progenitors of the newborn adrenal glands were reactive for NCAM, CD117, NSE, PDGFr-alpha, Synaptophysin, Hepatocyte growth factor and alpha-feto-protein. (et al., 2016) The outermost layer of the fetal and neonatal adrenal cortex, the zona glomerulosa, has been detected as the principal site of small clusters of undifferentiated adrenal stem/progenitor cells, that persist in this location throughout life. (Nishimoto et al., 2010) The presence of this adrenal stem/progenitors are probably at the basis of the continued proliferative capacity of the adult adrenal gland to maintain adrenal volume and function throughout life. (Walczak & Hammer, 2015) The discovery of developmental links between the fetal and the adult adrenal gland, and that the initial population of fetal adrenocortical cells contributes to the lineage of adult adrenal cells, (Zubair et al., 2008) makes the study of stem cells in the human newborn even more intriguing than in the past. Pbx1 and steroidogenic factor 1 (SF1) are considered two markers of adrenal stem/progenitor cells, and their expression is indispensable for adrenal gland differentiation and development (Schnabel et al., 2003) (Table 1).

#### Intestinal stem cells

Multiple markers have been utilized to identify stem/progenitor cells in the developing gut. The intestinal stem cells, also known as crypt base columnar cells (CBCCs), due to their location at the bottom of the intestinal crypts, are immunostained with antibodies for leucine-rich-repeat-containing G-protein-coupled receptor-5 (Lgr5). (Rodríguez-Colman et al., 2017) Signals of Wnt, BMP, Notch and EGF in the stem cell niche have a role in modulating the intestinal stem cell fate. (Qi & Chen, 2015) A Major role in the organization of the stem cell niche in the human gut is played by Paneth cells, that may be marked with anti-CD24 antibody. (Sato et al., 2011) Another marker expressed by human intestinal stem cells is WNT, Wnt signaling representing primarily the principal organizer of epithelial stem cell identity and proliferation. (Koch, 2017) Recently, casein kinase 1-epsilon and 1-delta have been shown to be necessary for the maintenance of the regenerative capacity of WNT-positive intestinal precursor cells. (Morgenstern et al., 2017) Notch signaling pathway has a major role in the homeostasis of the intestinal stem cell niche. (Sukhotnik et al., 2017) Other markers expressed by intestinal stem/progenitor cells are p-TEN, p-AKT, Fgfr3 and CD44 (Ambu et al., 2016) (Table 1).

#### Liver stem cells

The preterm liver represents a completely different organ as compared to the adult liver, due to the huge number of hemopoietic cells that characterize the liver in the intrauterine life. (Fanni et al., 2018) Another peculiar feature of the developing liver is represented by the immaturity of portal spaces which, during fetal life, probably represent the preferential site of the liver stem cell niches (Fig. 1f). (Fanni et al., 2016b) Stem/progenitor cells represent about 2% of fetal liver cells (Semeraro et al., 2013). Multiple types of liver stem cells have been identified. At the periphery of portal tracts, biliary progenitor cells originate to a double cylinder of small cells with oval nuclei, defined the ductal plate. These cells are characterized by the expression of CK19 and CK7, the two cytokeratins that characterize the biliary lineage. The multipotent undifferentiated hepatic stem/progenitors have been localized in the portal tracts. Their immunohistochemical profile is characterized by reactivity for NCAm, CD133, SOX9, SOX17, FOXA2, CK 8 and 18. The hepatoblasts are large cells with abundant cytoplasm, bordering the portal tracts. Their phenotypic profile overlaps with that of multipotent stem/progenitors, from which they differ for the strong expression of albumin and for expression of P450-A4 and A7. (Fanni et al., 2015a) (Fanni et al., 2015b) (Fanni et al., 2016c) Committed hepatic progenitors show albumin and glucose-6-phosphate expression, lacking CK19 and alpha-fetoprotein (Table 1).

#### Brain stem cells

The complexity of the human cerebral cortex is marked by the multiple progenitors involved in its development, each of them being characterized by the expression of multiple immunohistochemical markers. In the neonatal brain, stem/progenitors are located in the periventricular zone (Fig. 1 g). The most useful markers for the identification of cerebral stem cells have been recently summarized in a study from our group. (Vinci et al., 2016a) Nestin is one of the earliest markers expressed in the primary neuroepithelium, both in the ventricular and in the periventricular zone. (Murdoch & Roskams, 2008) Nestin also marks the cytoplasmic extensions of the radial glia that are directed towards the pial zone, probably representing a guide for migrating neuronal precursors. (Vinci et al., 2016b) SOX2 is one of the earliest markers expressed by brain stem cells during brain development, and it is considered a key factor in the induction of neurogenesis. (Zhang, 2014) In the fetal developing human cortex, SOX2 is expressed in the ventricular neuroepithelium and in migrating neurons, whereas its expression is down-regulated or absent in the post-mitotic neurons of the subpial zone. (Vinci et al., 2016b) Vimentin is expressed by multiple cell types in the developing cerebral cortex, including the radial glia. (Nakagawa et al., 2004) Another marker of radial glia cells is WT1, a transcription factor highly expressed in the human brain, as well as in multiple organs, during development (Ambu et al., 2015) (Table 1).

#### Endometrial stem cells

Stem cells have been observed to be abundant in the human endometrium. Endometrial stem cells are characterized by the following phenotype: CD44+, CD90+, CD105+, OCT4+. Endometrial stem progenitors have been reported to also express c-KIT (CD117), CD34 and bcl2. (Cho et al., 2004) A subset of endometrial mesenchymal stem cells are characterized by the co-expression of two perivascular cell markers, CD146 and platelet-derived growth factor-receptor beta (PDGF-Rbeta). (Co-expression of two perivascular cell markers isolates mesenchymal stem-like cells from human endometrium, 2020) A recent study based on flow cytometry, focused on the characterization of the markers of endometrial stem cells, indicated c, CD31, CD34, CD44, CD49d, CD54, CD73, CD90, CD104b, CD105, CD106, CD117 and CD166 as the typical markers of endometrial human stem cells. (Somasundaram, 2017) A recent study from our group identified ISL1 as a very useful tool for identifying endometrial stem cells in the developing human uterus. (Cau et al., 2019) Endometrial stem cells are pluripotent cells. When stimulated with specific signaling molecules, they may differentiate into multiple cell types, including neuronal cells. (Bardanzellu et al., 2018) According with these data, endometrial stem/progenitor cells might represent a unique source for regenerative cell therapy, in particular for neurodegenerative diseases (Mobarakeh et al., 2012) (Table 1).

## Conclusions

**Fig. 2:**
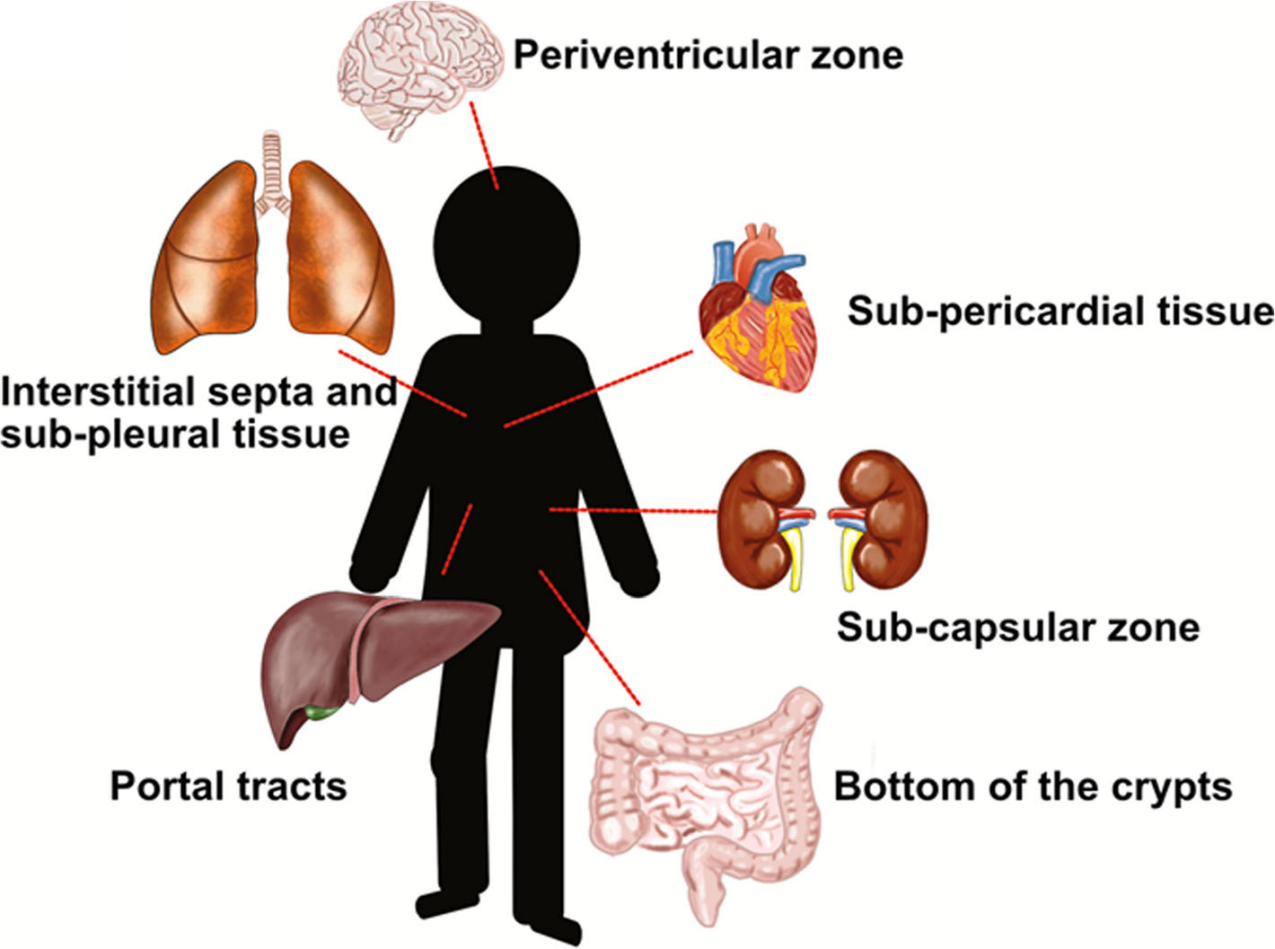
Schematic representation of the different localization of stem/progenitor cell niches in human organs during development.

The data here reported, clearly show that the fetal and preterm tissues represent a source of stem/progenitor cells. Their identification may be difficult, or impossible, when it is only based on morphology, due to the absence of differentiation, a typical feature of stemness. Immunohistochemistry allows the identification of stem cells in all organs, with some peculiarities in each organ and tissue (Table 1). Data from the literature, and for the personal experience of authors in this field, suggest that stem/progenitor cells express different markers in the different tissues. As a consequence, no general rule for their detection exists. In this review, the most important data regarding the useful markers for their identification in the fetal and preterm organs are reported. From a practical point of view, these data might be of some utility for pathologists involved in fetal and perinatal pathology. The study of stem cells in different organs might increase their ability in the interpretation of the physiopathology of preterm and at term newborns. Another reason makes the identification of stem/progenitor cells in fetal and perinatal tissues of some interest. Their abundance in multiple organs and tissues in the perinatal period might represent an incredible source of human cells with high regenerative potentials. This regenerative potential might be utilized for the regenerative medicine, the new field of human medicine with so many fascinating perspectives (Fig. 2). Further studies are needed in order to analyze the quantitative and qualitative changes of stem progenitor cells at different gestational ages in different organs, so as to shed light on the timeline of proliferation and differentiation of stem/progenitor cells in the prenatal/perinatal period.

## Data Availability

Not applicable.

## References

[CR1] Ahn S-H, Henderson YC, Williams MD, Lai SY, Clayman GL (2014). Detection of thyroid cancer stem cells in papillary thyroid carcinoma. J Clin Endocrinol Metab.

[CR2] Alatyyat SM, Alasmari HM, Aleid OA, Abdel-Maksoud MS, Elsherbiny N (2020). Umbilical cord stem cells: background, processing and applications. Tissue Cell.

[CR3] Alviano F, Fossati V, Marchionni C, Arpinati M, Bonsi L, Franchina M (2007). Term Amniotic membrane is a high throughput source for multipotent Mesenchymal Stem Cells with the ability to differentiate into endothelial cells in vitro. BMC Dev Biol.

[CR4] Ambu R, Gerosa C, Locci G, Obinu E, Ravarino A, De Magistris A, et al. The small intestinal mucosa and its stem cells. Vol. 5, Journal of Pediatric and Neonatal Individualized Medicine. Hygeia Press di Corridori Marinella; 2016. pag. e050224.

[CR5] Ambu R, Vinci L, Gerosa C, Fanni D, Obinu E, Faa A (2015). WT1 expression in the human fetus during development. Eur J Histochem.

[CR6] Angelotti ML, Ronconi E, Ballerini L, Peired A, Mazzinghi B, Sagrinati C, Parente E, Gacci M, Carini M, Rotondi M, Fogo AB, Lazzeri E, Lasagni L, Romagnani P (2012). Characterization of renal progenitors committed toward tubular lineage and their regenerative potential in renal tubular injury. Stem Cells.

[CR7] Antounians L, Tzanetakis A, Pellerito O, Catania VD, Sulistyo A, Montalva L (2019). The regenerative potential of amniotic fluid stem cell extracellular vesicles: lessons learned by comparing different isolation techniques. Sci Rep.

[CR8] Bardanzellu F, Faa G, Fanni D, Fanos V, Marcialis MA (2018). Regenerating the womb: the good, bad and ugly potential of the endometrial stem cells. Curr Regen Med.

[CR9] Bernardo AS, Cho CH-H, Mason S, Docherty HM, Pedersen RA, Vallier L (2009). Docherty K Biphasic induction of Pdx1 in mouse and human embryonic stem cells can mimic development of pancreatic β-cells. Stem Cells.

[CR10] Bertoncello I, McQualter JL (2013). Lung stem cells: do they exist?. Respirology.

[CR11] Bollini S, Cheung KK, Riegler J, Dong X, Smart N, Ghionzoli M, Loukogeorgakis SP, Maghsoudlou P, Dubé KN, Riley PR, Lythgoe MF, de Coppi P (2011). Amniotic fluid stem cells are cardioprotective following acute myocardial infarction. Stem Cells Dev.

[CR12] Cabuk D, Yetimoglu E, Simsek T, Gacar G, Subasi C, Canturk Z (2016). The distribution of CD44+/CD24- cancer stem cells in breast cancer and its relationship with prognostic factors. J BUON.

[CR13] Carraro G, Perin L, Sedrakyan S, Giuliani S, Tiozzo C, Lee J, Turcatel G, de Langhe SP, Driscoll B, Bellusci S, Minoo P, Atala A, de Filippo RE, Warburton D (2008). Human amniotic fluid stem cells can integrate and differentiate into epithelial lung lineages. Stem Cells.

[CR14] Carvalho AB (2010). Heart regeneration: past, present and future. World J Cardiol.

[CR15] Cau F, Botta MC, Vinci L, Gerosa C, Sorrentino E, Senes G (2019). ISL-1: A new potential marker of stem/progenitor cells in the developing human uterus. J Pediatr Neonatal Individualized Med.

[CR16] Cho NH, Park YK, Kim YT, Yang H, Kim SK (2004). Lifetime expression of stem cell markers in the uterine endometrium. Fertil Steril.

[CR17] Çil N, Oğuz EO, Mete E, Çetinkaya A, Mete G (2017). Effects of umbilical cord blood stem cells on healing factors for diabetic foot injuries. Biotech Histochem.

[CR18] Schwab KE, Gargett CE. Co-expression of two perivascular cell markers isolates mesenchymal stem-like cells from human endometrium. Hum Reprod. 2007;22(11):2903–11.10.1093/humrep/dem265. 10.1093/humrep/dem265 17872908

[CR19] Cregan MD, Fan Y, Appelbee A, Brown ML, Klopcic B, Koppen J, Mitoulas LR, Piper KME, Choolani MA, Chong YS, Hartmann PE (2007). Identification of nestin-positive putative mammary stem cells in human breastmilk. Cell Tissue Res.

[CR20] De Coppi P, Atala A. Stem cells from the amnion. In: principles of regenerative medicine [internet]. Elsevier; 2019 [citato 13 dicembre 2020]. Pag. 133–48. Disponibile su: https://linkinghub.elsevier.com/retrieve/pii/B9780128098806000096

[CR21] Li Y, Liu Z, Jin Y, Zhu X, Wang S, Yang J, Ren Y, Fu Q, Xiong H, Zou G, Liu Y. Differentiation of Human Amniotic Mesenchymal Stem Cells into Human Anterior Cruciate Ligament Fibroblast Cells by In Vitro Coculture. Biomed Res Int. 2017;2017:7360354. 10.1155/2017/7360354. 10.1155/2017/7360354 PMC5632453 29085840

[CR22] Eirew P, Stingl J, Raouf A, Turashvili G, Aparicio S, Emerman JT, Eaves CJ (2008). A method for quantifying normal human mammary epithelial stem cells with in vivo regenerative ability. Nat Med.

[CR23] Faa A, Podda E, Fanos V (2016). Stem cell markers in the heart of the human newborn. J Pediatr Neonatal Individualized Med.

[CR24] Faa G, Fanni D, Gerosa C, Fraschini M, Nemolato S, Ottonello G, et al. The Subcapsular Blue Strip Width: A New Marker for Evaluating the Residual Potential Nephrogenesis in the Newborn Kidney. 2013;

[CR25] Faa G, Gerosa C, Fanni D, Nemolato S, Di Felice E, Van Eyken P (2012). The role of immunohistochemistry in the study of the newborn kidney. J Matern Fetal Neonatal Medic.

[CR26] Faa G, Gerosa C, Fanni D, Nemolato S, Marinelli V, Locci A, Senes G, Mais V, Eyken PV, Iacovidou N, Monga G, Fanos V (2012). CD10 in the developing human kidney: Immunoreactivity and possible role in renal embryogenesis. J Matern Fetal Neonatal Med.

[CR27] Faa G, Sanna A, Gerosa C, Fanni D, Puddu M, Ottonello G, et al. Renal physiological regenerative medicine to prevent chronic renal failure: Should we start at birth? Vol. 444, Clinica Chimica Acta. Elsevier; 2015. pag. 156–62. 10.1016/j.cca.2015.02.023 25701508

[CR28] Fanni D, Fanos M, Gerosa C, Cau F, Pisu E, Van Eyken P (2016). Stem/progenitor cells in the developing human lung. J Pediatr Neonatal Individualized Med.

[CR29] Fanni D, Fanos V, Ambu R, Lai F, Gerosa C, Pampaloni P (2015). Overlapping between CYP3A4 and CYP3A7 expression in the fetal human liver during development. J Matern Fetal Neonatal Med.

[CR30] Fanni D, Fanos V, Gerosa C, Senes G, Sanna A, Van Eyken P (2013). CD44 immunoreactivity in the developing human kidney: a marker of renal progenitor stem cells?. Ren Fail.

[CR31] Fanni D, Fanos V, Monga G, Gerosa C, Locci A, Nemolato S (2011). Expression of WT1 during normal human kidney development. J Matern Fetal Neonatal Med.

[CR32] Fanni D, Fanos V, Monga G, Gerosa C, Nemolato S, Locci A (2011). MUC1 in mesenchymal-to-epithelial transition during human nephrogenesis: changing the fate of renal progenitor/stem cells?. J Matern Fetal Neonatal Med.

[CR33] Fanni D, Gerosa C, Lai F, Faa G. Human Hepatic Stem/Progenitor Cells in Cancer and Liver Disease. In: Pham P., El-Hashash A. (eds) Stem Cells for Cancer and Genetic Disease Treatment. Stem Cells in Clinical Applications. Cham: Springer; 2018. 10.1007/978-3-319-98065-2_5.

[CR34] Fanni D, Gerosa C, Lai F, Van Eyken P, Faa G (2016). Stem/progenitor cells in the developing human liver: Morphological and immunohistochemical features. J Pediatr Neonatal Individualized Med..

[CR35] Fanni D, Iacovidou N, Locci A, Gerosa C, Nemolato S, Van Eyken P (2012). MUC1 marks collecting tubules, renal vesicles, comma-and S-shaped bodies in human developing kidney. Eur J Histochem.

[CR36] Fanni D, Manchia M, Lai F, Gerosa C, Ambu R, Faa G (2016). Immunohistochemical markers of CYP3A4 and CYP3A7: a new tool towards personalized pharmacotherapy of hepatocellular carcinoma. Eur J Histochem.

[CR37] Fanni D, Sanna A, Gerosa C, Puddu M, Faa G, Fanos V. Each niche has an actor: multiple stem cell niches in the preterm kidney. Ital J Pediatr. 2015;41:78. 10.1186/s13052-015-0187-6. 10.1186/s13052-015-0187-6 PMC4608192 26472160

[CR38] Fanos V, Loddo C, Puddu M, Gerosa C, Fanni D, Ottonello G, Faa G. From ureteric bud to the first glomeruli: genes, mediators, kidney alterations. Int Urol Nephrol. 2015;47(1):109–16. 10.1007/s11255-014-0784-0. 10.1007/s11255-014-0784-0 25201458

[CR39] Farhadihosseinabadi B, Farahani M, Tayebi T, Jafari A, Biniazan F, Modaresifar K (2018). Amniotic membrane and its epithelial and mesenchymal stem cells as an appropriate source for skin tissue engineering and regenerative medicine. Artif Cells Nanomed Biotechnol.

[CR40] Fu N, Lindeman GJ, Visvader JE. The Mammary Stem Cell Hierarchy. In: Current Topics in Developmental Biology. Academic Press Inc.; 2014. pag. 133–160. 10.1016/B978-0-12-416022-4.00005-6 24439805

[CR41] Ge W, Cheng SF, Dyce PW, De Felici M, Shen W. Skin-derived stem cells as a source of primordial germ cell- and oocyte-like cells. Cell Death Dis. 2016;7(11):e2471. 10.1038/cddis.2016.366. 10.1038/cddis.2016.366 PMC5260893 27831564

[CR42] Gerosa C, Fanos V, Puddu M, Ottonello G, Faa G, Pinna B (2016). Not all renal stem cell niches are the same: Anatomy of an evolution. J Pediatr Neonatal Individualized Med.

[CR43] Harris D (2013). Umbilical cord tissue mesenchymal stem cells: characterization and clinical applications. Curr Stem Cell Res Ther.

[CR44] Hughes S (2002). Cardiac stem cells. J Pathol.

[CR45] Jaiswal RK, Jaiswal N, Bruder SP, Mbalaviele G, Marshak DR, Pittenger MF (2000). Adult human mesenchymal stem cell differentiation to the osteogenic or adipogenic lineage is regulated by mitogen-activated protein kinase. J Biol Chem.

[CR46] Kaingade P, Nikam A, Kulkarni S, et al. Marker profiles of human endometrial stem cells at various passages cultured in-vitro. J Stem Cell Res Ther. 2017;2(1):5–10. 10.15406/jsrt.2017.02.00051.

[CR47] Kiernan J, Damien P, Monaghan M, Shorr R, McIntyre L, Fergusson D, Tinmouth A, Allan D. Clinical Studies of Ex Vivo Expansion to Accelerate Engraftment After Umbilical Cord Blood Transplantation: A Systematic Review. Transfus Med Rev. 2017;31(3):173–82. 10.1016/j.tmrv.2016.12.004. 10.1016/j.tmrv.2016.12.004 28087163

[CR48] Kim Y, Jeong J, Kang H, Lim J, Heo J, Ratajczak J, Ratajczak MZ, Shin DM. The molecular nature of very small embryonic-like stem cells in adult tissues. Int J Stem Cells. 2014;7(2):55–62. 10.15283/ijsc.2014.7.2.55. 10.15283/ijsc.2014.7.2.55 PMC4249904 25473442

[CR49] Koch S. Extrinsic control of Wnt signaling in the intestine. Differentiation. 2017;97:1–8. 10.1016/j.diff.2017.08.003. 10.1016/j.diff.2017.08.003 28802143

[CR50] Laugwitz KL, Moretti A, Lam J, Gruber P, Chen Y, Woodard S (2005). Postnatal isl1+ cardioblasts enter fully differentiated cardiomyocyte lineages. Nature.

[CR51] Lee J-H, Hah Y-S, Cho H-Y, Kim J-H, Oh S-H, Park B-W, Kang YH, Choi MJ, Shin JK, Rho GJ, Jeon RH, Lee HC, Kim GC, Kim UK, Kim JR, Lee CI, Byun JH (2014). Human umbilical cord blood-derived CD34-positive endothelial progenitor cells stimulate osteoblastic differentiation of cultured human periosteal-derived osteoblasts. Tissue Eng Part A.

[CR52] Leyva-Leyva M, Barrera L, López-Camarillo C, Arriaga-Pizano L, Orozco-Hoyuela G, Carrillo-Casas EM (2013). Characterization of mesenchymal stem cell subpopulations from human amniotic membrane with dissimilar osteoblastic potential. Stem Cells Dev.

[CR53] Liu J, Brown RE (2010). Immunohistochemical detection of epithelialmesenchymal transition associated with stemness phenotype in anaplastic thyroid carcinoma. Int J Clin Exp Pathol.

[CR54] Lloyd-Griffith C, McFadden TM, Duffy GP, Unger RE, Kirkpatrick CJ, O’Brien FJ (2015). The pre-vascularisation of a collagen-chondroitin sulphate scaffold using human amniotic fluid-derived stem cells to enhance and stabilise endothelial cell-mediated vessel formation. Acta Biomater.

[CR55] Locci G, Pinna AP, Dessì A, Obinu E, Gerosa C, Marcialis MA (2016). Stem progenitor cells in the human pancreas. J Pediatr Neonatal Individualized Med.

[CR56] Mahalingam M, Nguyen LP, Richards JE, Muzikansky A, Hoang MP (2010). The diagnostic utility of immunohistochemistry in distinguishing primary skin adnexal carcinomas from metastatic adenocarcinoma to skin: an immunohistochemical reappraisal using cytokeratin 15, nestin, p63, D2-40, and calretinin. Modern Pathol.

[CR57] Martin CM, Meeson AP, Robertson SM, Hawke TJ, Richardson JA, Bates S (2004). Persistent expression of the ATP-binding cassette transporter, Abcg2, identifies cardiac SP cells in the developing and adult heart. Dev Biol.

[CR58] Matsuura K, Nagai T, Nishigaki N, Oyama T, Nishi J, Wada H (2004). Adult Cardiac Sca-1-positive Cells Differentiate into Beating Cardiomyocytes. J Biol Chem.

[CR59] Miki T, Lehmann T, Cai H, Stolz DB, Strom SC (2005). Stem cell characteristics of amniotic epithelial cells. Stem Cells.

[CR60] Miki T, Mitamura K, Ross MA, Stolz DB, Strom SC (2007). Identification of stem cell marker-positive cells by immunofluorescence in term human amnion. J Reprod Immunol.

[CR61] Mobarakeh ZT, Ai J, Yazdani F, Sorkhabadi SMR, Ghanbari Z, Javidan AN (2012). Human endometrial stem cells as a new source for programming to neural cells. Cell Biol Int Rep.

[CR62] Monz D, Tutdibi E, Gortner L. Stem cells as therapeutical option for the treatment of bronchopulmonary dysplasia. J Pediatr Neonat Individual Med. 2015;5(1):e050116.

[CR63] Morgenstern Y, Das Adhikari U, Ayyash M, Elyada E, Tóth B, Moor A (2017). Casein kinase 1-epsilon or 1-delta required for Wnt-mediated intestinal stem cell maintenance. EMBO J.

[CR64] Murdoch B, Roskams AJ (2008). A novel embryonic nestin-expressing radial glia-like progenitor gives rise to zonally restricted olfactory and vomeronasal neurons. J Neurosci.

[CR65] Nakagawa T, Miyazaki T, Miyamoto O, Janjua NA, Hata T, Itano T (2004). Regional expression of the radial glial marker vimentin at different stages of the kindling process. Epilepsy Res.

[CR66] Nemolato S, Cabras T, Messana I, Gerosa C, Faa G, Castagnola M. Do β-Thymosins Play a Role in Human Nephrogenesis? In Humana Press, New York, NY; 2014. pag. 81–93.

[CR67] Nishimoto K, Nakagawa K, Li D, Kosaka T, Oya M, Mikami S, Shibata H, Itoh H, Mitani F, Yamazaki T, Ogishima T, Suematsu M, Mukai K (2010). Adrenocortical zonation in humans under normal and pathological conditions. J Clin Endocrinol Metab.

[CR68] Nostro MC, Sarangi F, Ogawa S, Holtzinger A, Corneo B, Li X (2011). Stage-specific signaling through TGFβ family members and WNT regulates patterning and pancreatic specification of human pluripotent stem cells. Development.

[CR69] Oh H, Bradfute SB, Gallardo TD, Nakamura T, Gaussin V, Mishina Y (2003). Cardiac progenitor cells from adult myocardium: Homing, differentiation, and fusion after infarction. Proc Nat Acad Sci U S A.

[CR70] Parolini O, Alviano F, Bagnara GP, Bilic G, Bühring H-J, Evangelista M, Hennerbichler S, Liu B, Magatti M, Mao N, Miki T, Marongiu F, Nakajima H, Nikaido T, Portmann-Lanz CB, Sankar V, Soncini M, Stadler G, Surbek D, Takahashi TA, Redl H, Sakuragawa N, Wolbank S, Zeisberger S, Zisch A, Strom SC (2008). Concise review: isolation and characterization of cells from human term placenta: outcome of the first international workshop on placenta derived stem cells. Stem Cells.

[CR71] Perin L, Giuliani S, Jin D, Sedrakyan S, Carraro G, Habibian R, Warburton D, Atala A, de Filippo RE (2007). Renal differentiation of amniotic fluid stem cells. Cell Prolif.

[CR72] Pichiri G, Lanzano D, Piras M, Dessì A, Reali A, Puddu M, Noto A, Fanos V, Coni C, Faa G, Coni P. Human breast milk stem cells: a new challenge for perinatologists. J Pediatr Neonat Individual Med. 2016;5(1):e050120.

[CR73] Qi Z, Chen YG. Regulation of intestinal stem cell fate specification. Vol. 58, Science China Life Sciences. Science in China Press; 2015. pag. 570–8. 10.1007/s11427-015-4859-7 25951932

[CR74] Ratajczak MZ, Shin DM, Liu R, Mierzejewska K, Ratajczak J, Kucia M, Zuba-Surma EK. Very small embryonic/epiblast-like stem cells (VSELs) and their potential role in aging and organ rejuvenation--an update and comparison to other primitive small stem cells isolated from adult tissues. Aging (Albany NY). 2012;4(4):235–46. 10.18632/aging.100449. 10.18632/aging.100449 PMC3371759 22498452

[CR75] Rios AC, Fu NY, Lindeman GJ, Visvader JE (2014). In situ identification of bipotent stem cells in the mammary gland. Nature.

[CR76] Rodríguez-Colman MJ, Schewe M, Meerlo M, Stigter E, Gerrits J, Pras-Raves M (2017). Interplay between metabolic identities in the intestinal crypt supports stem cell function. Nature.

[CR77] Romanov YA, Svintsitskaya VA, Smirnov VN. Searching for alternative sources of postnatal human mesenchymal stem cells: candidate MSC-like cells from umbilical cord. Stem Cells. 2003;21(1):105–10. 10.1634/stemcells.21-1-105. 10.1634/stemcells.21-1-105 12529557

[CR78] Ruiz-Babot G, Hadjidemetriou I, King PJ, Guasti L. New directions for the treatment of adrenal insufficiency. Front Endocrinol (Lausanne). 2015;6:70. 10.3389/fendo.2015.00070. 10.3389/fendo.2015.00070 PMC4422080 25999916

[CR79] Sanna A, Fanos V, Gerosa C, Vinci L, Puddu M, Loddo C (2015). Immunohistochemical markers of stem/progenitor cells in the developing human kidney. Acta Histochem.

[CR80] Sato T, Van Es JH, Snippert HJ, Stange DE, Vries RG, Van Den Born M (2011). Paneth cells constitute the niche for Lgr5 stem cells in intestinal crypts. Nature.

[CR81] Schmelzer E, McKeel DT, Gerlach JC. Characterization of Human Mesenchymal Stem Cells from Different Tissues and Their Membrane Encasement for Prospective Transplantation Therapies. Biomed Res Int. 2019;2019:6376271. 10.1155/2019/6376271. 10.1155/2019/6376271 PMC6421008 30941369

[CR82] Schnabel CA, Selleri L, Cleary ML (2003). Pbx1 is essential for adrenal development and urogenital differentiation. Genesis.

[CR83] Semeraro R, Cardinale V, Carpino G, Gentile R, Napoli C, Venere R, et al. The fetal liver as cell source for the regenerative medicine of liver and pancreas. Ann Transl Med. 2013;1(2). 10.3978/j.issn.2305-5839.2012.10.02 PMC4200630 25332958

[CR84] Seymour PA, Freude KK, Tran MN, Mayes EE, Jensen J, Kist R (2007). SOX9 is required for maintenance of the pancreatic progenitor cell pool. Proc Natl Acad Sci U S A.

[CR85] Shimasue A, Yamakawa N, Watanabe M, Hidaka Y, Iwatani Y, Takano T (2015). Expression analysis of stemness genes in a rat thyroid cell line FRTL5. Exp Clin Endocrinol Diabetes.

[CR86] Soncini M, Vertua E, Gibelli L, Zorzi F, Denegri M, Albertini A, Wengler GS, Parolini O (2007). Isolation and characterization of mesenchymal cells from human fetal membranes. J Tissue Eng Regen Med.

[CR87] Srivastava M, Ahlawat N, Srivastava A (2018). Amniotic fluid stem cells: a new era in regenerative medicine. J Obstet Gynecol India.

[CR88] Sukhotnik I, Coran AG, Pollak Y, Kuhnreich E, Berkowitz D, Saxena AK (2017). Activated notch signaling cascade is correlated with stem cell differentiation toward absorptive progenitors after massive small bowel resection in a rat. Am J Physiol Gastrointest Liver Physiol.

[CR89] Sun Y, Kong W, Huang S, Shi B, Zhang H, Chen W (2017). Comparable therapeutic potential of umbilical cord mesenchymal stem cells in collagen-induced arthritis to TNF inhibitor or anti-CD20 treatment. Clin Exp Rheumatol.

[CR90] Takano T (2014). Fetal cell carcinogenesis of the thyroid: a modified theory based on recent evidence. Endocr J.

[CR91] Thomas T, Nowka K, Lan L, Derwahl M (2006). Expression of endoderm stem cell markers: evidence for the presence of adult stem cells in human thyroid glands. Thyroid.

[CR92] Torrente Y, Tremblay JP, Pisati F, Belicchi M, Rossi B, Sironi M (2001). Intraarterial injection of muscle-derived CD34+Sca-1+ stem cells restores dystrophin in mdx mice. J Cell Biol.

[CR93] Twigger AJ, Hepworth AR, Lai CT, Chetwynd E, Stuebe AM, Blancafort P, Hartmann PE, Geddes DT, Kakulas F. Gene expression in breastmilk cells is associated with maternal and infant characteristics. Sci Rep. 2015;5:12933. 10.1038/srep12933. 10.1038/srep12933 PMC4542700 26255679

[CR94] Twigger AJ, Hodgetts S, Filgueira L, Hartmann PE, Hassiotou F (2013). From breast milk to brains: the potential of stem cells in human milk. J Hum Lact.

[CR95] Twigger AJ, Hodgetts S, Filgueira L, Hartmann PE, Hassiotou F. From breast milk to brains: the potential of stem cells in human milk. J Hum Lact. 2013;29(2):136-9. 10.1177/0890334413475528. 10.1177/0890334413475528 23515086

[CR96] Van Keymeulen A, Rocha AS, Ousset M, Beck B, Bouvencourt G, Rock J (2011). Distinct stem cells contribute to mammary gland development and maintenance. Nature.

[CR97] Vinci L, Ravarino A, Fanos V, Naccarato AG, Senes G, Gerosa C (2016). Immunohistochemical markers of neural progenitor cells in the early embryonic human cerebral cortex. Eur J Histochem.

[CR98] Vinci L, Ravarino A, Gerosa C, Pintus MC, Marcialis MA, Marinelli V, Faa G, Fanos V, Ambu R. Stem/progenitor cells in the cerebral cortex of the human preterm: a resource for an endogenous regenerative neuronal medicine? J Pediatr Neonat Individual Med. 2016;5(1):e050121. 10.7363/050121.

[CR99] Visvader JE, Stingl J. Mammary stem cells and the differentiation hierarchy: current status and perspectives. Genes Dev. 2014;28(11):1143–58. 10.1101/gad.242511.114. 10.1101/gad.242511.114 PMC4052761 24888586

[CR100] Walczak EM, Hammer GD. Regulation of the adrenocortical stem cell niche: implications for disease. Nat Rev Endocrinol. 2015;11(1):14–28. 10.1038/nrendo.2014.166. 10.1038/nrendo.2014.166 PMC4648246 25287283

[CR101] Wandzioch E, Zaret KS (2009). Dynamic signaling network for the specification of embryonic pancreas and liver progenitors. Science.

[CR102] Wu D, Zou S, Chen H, Li X, Xu Y, Zuo Q, Pan Y, Jiang SW, Huang H, Sun L (2017). Transplantation routes affect the efficacy of human umbilical cord mesenchymal stem cells in a rat GDM model. Clin Chim Acta.

[CR103] Zabierowski SE, Herlyn M. Melanoma stem cells: the dark seed of melanoma. J Clin Oncol. 2008;26(17):2890–4. 10.1200/JCO.2007.15.5465. 10.1200/JCO.2007.15.5465 18539969

[CR104] Zakrzewski W, Dobrzyński M, Szymonowicz M, Rybak Z. Stem cells: past, present, and future. Stem Cell Res Ther. 2019;10(1):68. 10.1186/s13287-019-1165-5. 10.1186/s13287-019-1165-5 PMC6390367 30808416

[CR105] Zani A, Cananzi M, Fascetti-Leon F, Lauriti G, Smith VV, Bollini S, Ghionzoli M, D'Arrigo A, Pozzobon M, Piccoli M, Hicks A, Wells J, Siow B, Sebire NJ, Bishop C, Leon A, Atala A, Lythgoe MF, Pierro A, Eaton S, de Coppi P (2014). Amniotic fluid stem cells improve survival and enhance repair of damaged intestine in necrotising enterocolitis via a COX-2 dependent mechanism. Gut.

[CR106] Zhang C, Yu L, Liu S, Wang Y. Human amnion-derived mesenchymal stem cells promote osteogenic and angiogenic differentiation of human adipose-derived stem cells. PLoS One. 2017;12(10). 10.1371/journal.pone.0186253 PMC5636128 29020045

[CR107] Zhang S (2014). Sox2, a key factor in the regulation of pluripotency and neural differentiation. World J Stem Cells.

[CR108] Zhou B, Ma Q, Rajagopal S, Wu SM, Domian I, Rivera-Feliciano J (2008). Epicardial progenitors contribute to the cardiomyocyte lineage in the developing heart. Nature.

[CR109] Zhou Q, Law AC, Rajagopal J, Anderson WJ, Gray PA, Melton DA (2007). A Multipotent Progenitor Domain Guides Pancreatic Organogenesis. Dev Cell.

[CR110] Zubair M, Parker KL, Morohashi K (2008). Developmental Links between the Fetal and Adult Zones of the Adrenal Cortex Revealed by Lineage Tracing. Mol Cell Biol.

[CR111] Obinu E, Locci G, Gerosa C, Fanos V, Vinci L, Faa G, et al. Adrenal stem cell niches are located between adrenal and renal capsules. J Pediatr Neonatal Individualized Med 2016;5(2):e050214.

